# Supplementary figures and images for: Functional Whole Genome Screen of Nutrient-Starved Mycobacterium tuberculosis Identifies Genes Involved in Rifampin Tolerance
Source: Microorganisms. 2023 Sep 9;11(9):2269. doi: 10.3390/microorganisms11092269 (PMC10534295; doi:10.3390/microorganisms11092269)

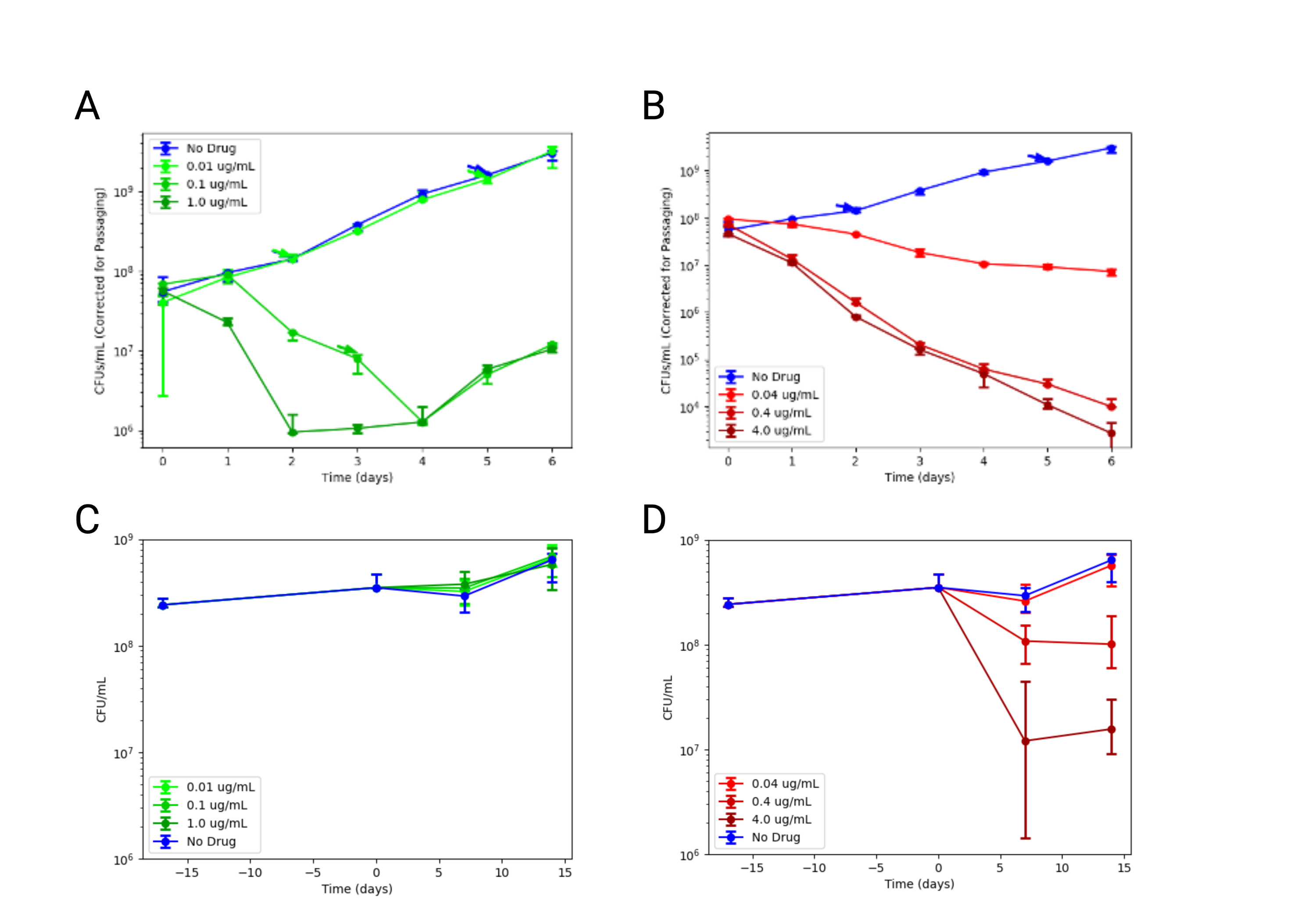

Supplement: Supplementary file 1 [file microorganisms-11-02269-s001.zip › microorganisms-2575757-supplementary/Supplementary Data/Supplemental Fig 1 (1).png]

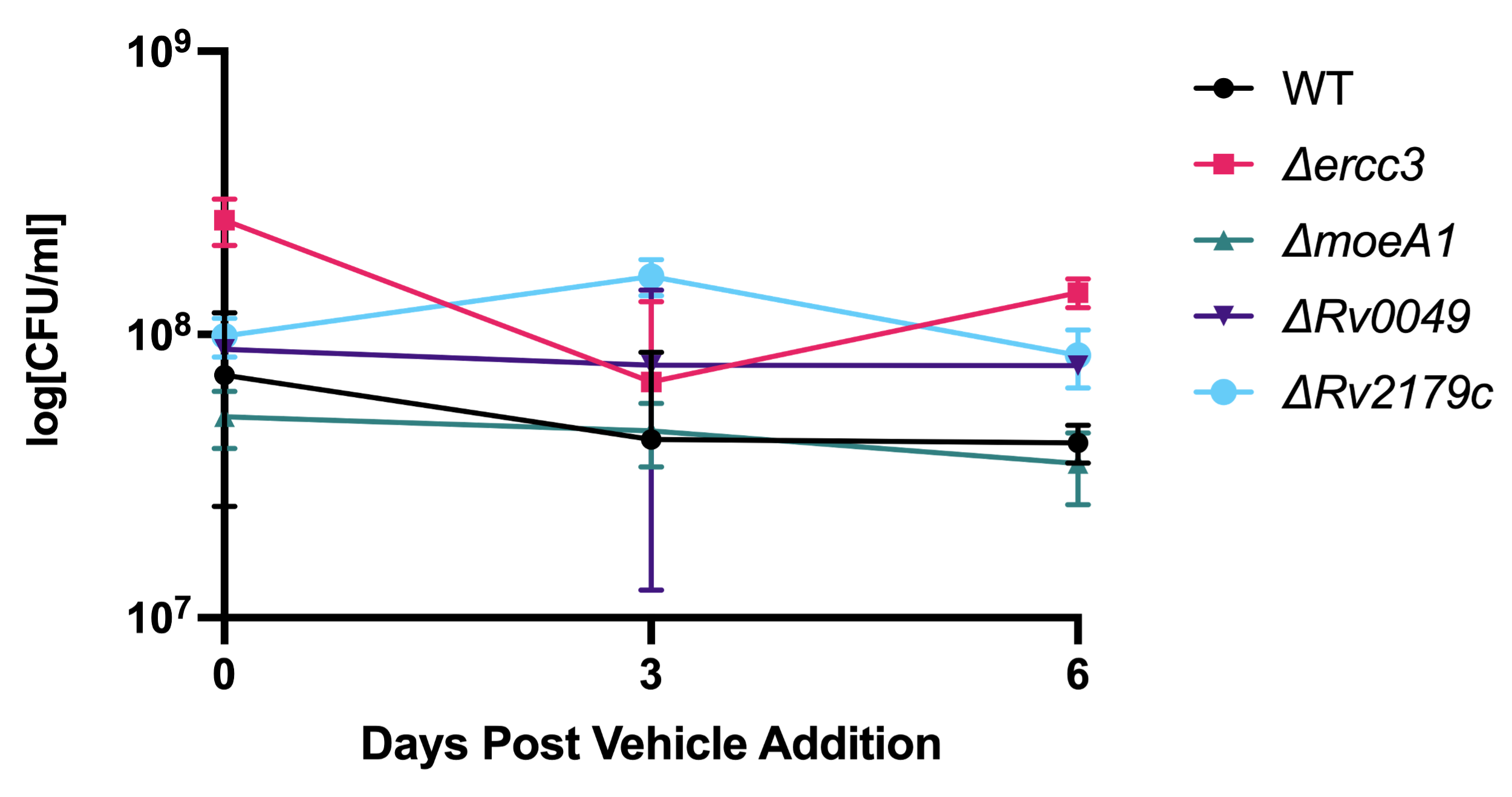

Supplement: Supplementary file 1 [file microorganisms-11-02269-s001.zip › microorganisms-2575757-supplementary/Supplementary Data/Supplemental Fig 2.png]
